# Bone mineral density and incidence of hip fracture in Swedish urban and rural women 1987–2002

**DOI:** 10.3109/17453674.2010.492762

**Published:** 2010-07-16

**Authors:** Björn E Rosengren, Henrik G Ahlborg, Per Gärdsell, Ingemar Sernbo, Robin M Daly, Jan-Åke Nilsson, Magnus K Karlsson

**Affiliations:** ^1^Clinical and Molecular Osteoporosis Research Unit, Department of Clinical Sciences and Department of Orthopaedics, SUS Malmö, Lund University; MalmöSweden; ^2^Department of Medicine, University of Melbourne, Western Hospital, MelbourneAustralia

## Abstract

**Background and purpose:**

Although the incidence of hip fracture during the past 50 years has increased, a break in this trend has been reported in the last decade. Whether this change is attributable to changes in bone mineral density (BMD) or whether it varies between urban and rural regions is unknown.

**Methods:**

We evaluated changes in annual hip fracture incidence in women aged ≥ 50 years in one urban population (n = 51,757) and one rural population (n = 26,446) from 1987 to 2002. We also examined secular differences in BMD (mg/cm^2^), evaluated by single-photon absorptiometry at the distal radius, prevalence of osteoporosis, and several other risk factors for hip fracture in one population-based sample of urban women and one sample of rural women aged 50–80 years at two time points: 1988/89 (n = 257 and n = 180, respectively) and 1998/99 (n = 171 and n = 118, respectively).

**Results:**

No statistically significant changes were evident in annual age-adjusted hip fracture incidence per 10^4^ when analyzing all women (–0.01 per year (95% CI: –0.37, 0.35)), rural women (–0.38 per year (-1.05, 0.28)), or urban women (0.19 per year (–0.28, 0.67)). BMD (expressed as T-score) was similar in 1988/99 and 1998/99 when analyzing all women (–0.09 (–0.26, 0.09)), urban women (–0.04 (–0.27, 0.19)), or rural women (–0.15 (–0.42, 0.13)) women.

**Interpretation:**

Since no changes in age-adjusted hip fracture incidence and no differences in BMD were found during the study period, changes evident in the other risk factors for hip fracture that we investigated (such as gait velocity and balance) are either of minor importance or are counteracted by changes in other risk factors.

## Introduction

An increase in hip fracture incidence during the last half-century has been reported worldwide, and in many reports this has been predicted to continue (Cooper et al. 1992, Gullberg et al. 1997, Kannus et al. 1999). Recent publications have opposed this view; the incidence of hip fracture in many western societies now seems to be stable or even decreasing (Melton et al. 1996, Rogmark et al. 1999, Lofman et al. 2002, Jaglal et al. 2005, Kannus et al. 2006, Nymark et al. 2006, Chevalley et al. 2007, Icks et al. 2008). Some reports have suggested that the incidence of hip fracture is higher in urban settings than in rural settings (Mannius et al. 1987, Sernbo et al. 1988, Jonsson et al. 1992, Melton et al. 1999, Chevalley et al. 2002, Sanders et al. 2002), at least partly due to a group discrepancy in bone mineral density (BMD) (Gardsell et al. 1991, Sundberg et al. 1997, Meyer et al. 2004, Pongchaiyakul et al. 2005, Gu et al. 2007). To our knowledge, there have been no studies evaluating whether these changes in hip fracture incidence are associated with changes in BMD or whether the changes are similar in urban and rural settings.

This observational study was designed to evaluate (i) whether there has been a decrease in hip fracture incidence in Swedish urban and rural women, and (ii) whether changes in hip fracture incidence could be attributed to changes in BMD or prevalence of osteoporosis. A secondary investigation was also done, examining differences in other known risk factors for hip fracture.

## Patients and methods

### Hip fractures

For fracture evaluation, we included the entire female population aged 50 years or more of (i) the city of Malmö, representing an urban region, and (ii) 9 municipalities near the county village of Sjöbo (Sjöbo, Tomelilla, Simrishamn, Bromölla, Skurup, Hörby, Höör, Ystad, and Osby), representing a rural region, in the period from 1987 to 2002. The populations of the urban and rural regions were determined using the national population records. In 1987, the population of the urban area was 230,838 and 51,757 (22%) were women aged 50 or above; in the rural area the population was 134,458 and 26,446 (20%) were women aged 50 or above. In 2002 the population of the urban area was 265,481 and 51,169 (19%) were women aged 50 or above; in the rural area the population was 141,989 and 30,391 (21%) were women aged 50 or above. The number of hip fractures was obtained from the register of the National Board of Health and Welfare, which includes all patients discharged from hospital in Sweden with data classified according to the disease treated and the surgical procedure. We selected patients who were classified as having an acute proximal femoral fracture by the diagnosis code ICD9 820x, ICD10, S720, S721, or S722 in the diagnosis code fields and with a surgical procedure for proximal femur fracture by the operation code ICD9 841,82x or ICD10 NFB, NFJ in the operation code fields.

### Primary and secondary investigations

**Figure 1.**
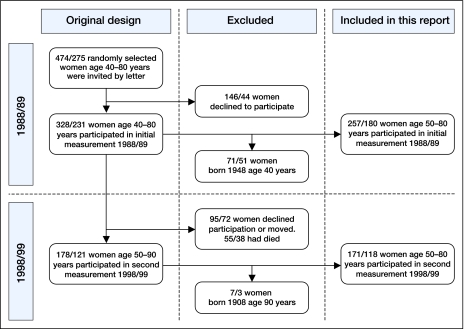
Enrollment and participation of urban and rural women in the study.

474 Caucasian women, born in 1908 (aged 80), 1918 (aged 70), 1928 (aged 60), 1938 (aged 50), and 1948 (aged 40) and living in the city of Malmö, and 275 age-matched Caucasian women living in the rural municipality of Sjöbo in Sweden were randomly selected from the national population records for 1988. These women were invited to participate in a prospective, population-based study mainly for evaluation of bone mineral density BMD but also of other lifestyle and neuromuscular functions (Gärdsell et al. 1991). In 1998, all previously measured women were invited by letter to a follow-up evaluation, giving a mean follow-up period of 9.6 (9.0–10.8) years in the urban cohort and 9.6 (9.3–10.3) years in the rural cohort (Figure 1 and Table 1).

**Table 1.** Number of participant in the study by age, cohort and measurement and mean age per cohort

	1988/89		1998/99	
Age	Urban	Rural	Urban	Rural
50	63	46	53	40
60	60	46	41	29
70	85	53	41	29
80	49	35	36	20
Total number	257	180	171	118
Mean age (years)	65	64	65	62
(95% CI)	(64–67)	(62–66)	(63–66)	(61–64)

The urban area, Malmö, is located in southern Sweden, and with its 231,575 (1988) and 254,904 (1998) residents, it is the third largest city in the country. The rural region, Sjöbo, is located 60 km east of Malmö in a farming district, and with 15,350 (1988) and 16,606 (1998) residents it represents a Swedish rural population.

Based on these measurements, we defined 4 population-based samples of women aged 50–80 years at measurement: (i) urban women measured in 1988/89 (n = 257), (ii) urban women measured in 1998/99 (n = 171), (iii) rural women measured in 1988/89 (n = 180), and (iv) rural women measured in 1998/99 (n = 118) (Figure 1 and Table 1).

Bone mineral density (BMD, mg/cm^2^) was measured in the writing forearm at 6 cm (BMD 6 cm) proximal to the ulnar styloid process by single-photon absorptiometry (SPA). The technique involves a rectilinear scan across the radius and ulna, with the radiation source (241 Am) and detector moving simultaneously, according to the description by Nauclér et al. (1974). The same densitometer was used throughout the study and the same technician analyzed all plots. The long-term drift was 0.1% per year (95% CI: –0.2, 0.4), evaluated by a standardized phantom every second week during the 10-year period. The coefficient of variation was 1–2% when evaluated by the phantom and 4% when evaluated by repeated measurement after repositioning of the arm in 20 subjects (Nauclér et al. 1974). Osteoporosis was defined by WHO criteria as a BMD value lower than 2.5 SD below the mean of a young reference population (WHO 1994). T-score was defined as the actual BMD value in relation to the mean and SD of a cohort of healthy young individuals. The values for definition of both osteoporosis and T-score were derived from a non-population-based sample of 38 healthy women aged 20–39 years measured in 1971 using the same equipment as in the present study with a mean BMD of 542 (SD 76) mg/cm^2^. Weight and height were determined in 1988/89 using a questionnaire and in 1998/99 by measurement with electronic scales and a standard height meter, and the values were used for calculation of BMI.

Medical history, including the presence of chronic diseases (diabetes mellitus, heart disease, lung disease, stroke, thyroid disease, epilepsy, rheumatic disease, and Parkinson's disease), use of certain medications (corticosteroids, thyroid, diabetic (including insulin)), smoking habits (non-smoker, former smoker, or current smoker), use of oral contraceptives (never vs. current or former) and estrogen therapy (never vs. current or former), age at menarche and menopause, history of oophorectomy, alcohol consumption, disability, dizziness (no, every week, every day), falling (never, occasionally, once or twice a month, on a weekly basis) and subjective health (good, fairly good, poor, very poor) were evaluated using the same questionnaire at both measurements. Participants were classified as having chronic disease(s) or medication use if they answered “yes” to any of the diseases or medications listed above. For those who reported not being teetotallers, the average intake of beer, wine, and hard liquor was used to estimate grams of alcohol consumed per week. Disability was defined as having difficulty in performing common activities of daily living, and was assessed by asking the participants whether they required outside assistance to perform daily activities (e.g., shopping, dishwashing, cleaning, personal hygiene), or could not manage activities such as shopping, dressing, making their bed, or going to the toilet. If they answered “yes” to any of these questions, they were classified as being physically disabled. Menopause was defined as occurring 1 year after the last menstrual period or at the time of oophorectomy. Gait velocity and balance were evaluated objectively as previously described (Ringsberg et al. 1998).

### Statistics

All continuous variables were found to be normally distributed by analysis by Shapiro Wilks test for normality, apart from age, which was almost normally distributed and accepted for parametric testing with Student's t-test due to the central limit theorem, and alcohol consumption per week, which was not normally distributed. For continuous normally distributed variables, Student's t-test was used. For binominal variables, chi-squared test was used and for variables with more than 2 categories and for non-normally distributed continuous variables, the Mann-Whitney U-test was used when comparing the groups. Data are presented as means with changes or differences together with 95% confidence intervals in parentheses unless otherwise specified. Age-adjustment for fractures was done in 1-year classes by direct standardization with the population of 1987/88 as the standard population. Time-trend analysis of secular changes in hip fracture incidence was done by linear regression. For the primary investigation, we only included analysis of BMD, BMI, and osteoporosis when comparing urban women in 1988/89 to urban women in 1998/99 and rural women in 1988/89 to rural women in 1998/99. Since there is a high correlation between BMD and osteoporosis, a Bonferroni correction factor of 2 × 2 was chosen in the primary analysis. For the secondary survey including 20 variables, a Bonferroni factor of 20 × 2 was chosen. Statistical calculations were performed with SPSS software version 15.0 and Statistica software version 7.1. For database handling, SAS system version 9.1 was used.

### Ethics

The study was approved by the Research Ethics Committee (ERC) (LU 258-88, September 14, 1988; LU 208-98, May 13, 1998) and carried out in accordance with the Helsinki Declaration.

## Results

### Hip fracture incidence

In the rural area, 3,175 hip fractures occurred in women aged 50 years or more during the study period, with 186 fractures in 1987 and 185 fractures in 2002 (corresponding to a an age-adjusted hip fracture incidence of 71 per 10^4^ and 60 per 10^4^). During the study period, there was a mean of 198 (SD 18) fractures per year and a mean age-adjusted hip fracture incidence of 68 (SD 6) per 10^4^ women per year.

In the urban area, 6,690 hip fractures occurred in women aged 50 years or more during the study period from 1987 to 2002, with 383 fractures in 1987 and 424 fractures in 2002 (corresponding to an age-adjusted hip fracture incidence of 75 per 10^4^ and 69 per 10^4^). During the study period, there was a mean of 418 (SD 39) fractures per year and a mean age-adjusted hip fracture incidence of 73 (SD 4) per 10^4^ women per year.

**Figure 2.**
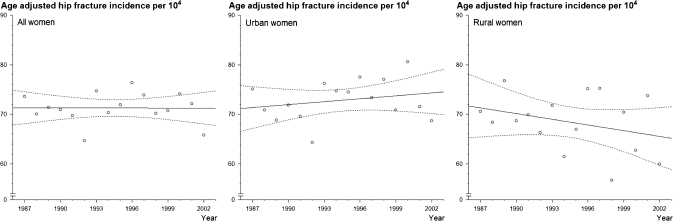
Annual age-adjusted hip fracture incidence between 1987 and 2002 in all women, urban women, and rural women (aged ≥ 50 years), with 95% confidence interval. There were no statistically significant changes in any group.

During the study period, there was no statistically significant change in the age-adjusted hip fracture incidence (number of hip fractures per 10^4^ women per year) when including all women (–0.01 per year (–0.37, 0.35)), rural women (–0.38 per year (–1.1, 0.28)), or urban women (0.19 per year (–0.28, 0.67)) (Figure 2).

When comparing the municipality of Sjöbo, where the BMD data were collected, to the 8 surrounding rural municipalities, no statistically significant differences were seen regarding mean age-adjusted hip fracture incidence (–4.5 per 10^4^ (–14, 5)) or changes in the age-adjusted hip fracture incidence (0.16 per 10^4^ per year (–0.86, 1.19)) during the study period.

### Bone mineral density, osteoporosis, BMI, and secondary investigation

BMD, prevalence of osteoporosis, and BMI were similar when we compared measurements of 1988/89 to those of 1998/99 whether including all women, only the urban women, or only the rural women (Table 2). In summary, the secondary investigation regarding 20 different lifestyle and neuromuscular variables showed that at the second measurement there was better balance, higher gait velocity, and a higher proportion of women who consumed alcohol (Table 3).

**Table 2.** Primary investigation of the urban and rural female study participants in 1988/89 and 1998/99

	Urban		Rural	
	1988/89	1998/99	1988/89	1998/99
	n = 257	n = 171	n = 180	n = 118
BMD (mg/cm^2^)	446 (436–457)	450 (435–464)	460 (447–472)	471 (453–488)
Prevalence of osteoporosis (%)	15 (11–21)	17 (11–23)	10 (6–16)	11 (6–18)
BMI (kg/m^2^)	25 (25–26)	26 (25–27)	28 (27–28)	27 (26–27)

Data are presented as mean (95% CI). Analysis of the women in the same area in 1988–1989 and 1998–1999 showed no significant differences after Bonferroni correction.

**Table 3.** Secondary investigation of the urban and rural female study participants in 1988/89 and 1998/99

	Urban		Rural	
	1988/89	1998/99	1988/89	1998/99
	n = 257	n = 171	n = 180	n = 118
Height (cm)	162 ± 6	164 ± 7	161 ± 6	163 ± 6
Weight (kg)	67 ± 11	69 ± 13	71 ± 13	70 ± 14
Age at menarche (years)	14 ± 2	14 ± 2	14 ± 2	14 ± 1
Menopause, n (%)	229 (89)	137 ± 80	148 ± 82	89 ± 75
Age at menopause (years)	49 ± 4	49 ± 4	49 ± 5	49 ± 4
Years postmenopausal	18 ± 11 ^**a**^	15 ± 12	19 ± 10 ^**a**^	13 ± 11
Estrogen therapy (%)	^**a**^		^**a**^	
Never	90	66	93	77
Former/Current	10	35	7	23
Oral contraceptives (%)	^**a**^		^**a**^	
Never	91	79	89	70
Former/Current	9	21	11	31
Cortico steroid therapy (%)				
Never	94	88	94	89
Former/Current	6	12	6	11
Dizziness/instability (%)				
No	81	83	80	90
Every week	11	14	16	9
Every day	8	2	4	2
Falling (%)				
Never	86	85	88	95
Occasionally	13	13	11	4
1–2 falls/month	2	2	1	1
Weekly basis	0	0	0	0
Smoking (%)			^**a**^	
Never	67	57	75	55
Former	12	19	10	25
Current	21	24	15	21
Teetotaller (%)	50	37	72 ^**a**^	46
Alcohol (g/week)	38 (23–-61)	38 (15–-62)	23 (15–-62)	41 (24–-61)
History of disease/medication use (%)	39	42	34	37
Self-reported disability (%)	19	11	15	6
Self-reported health (%)				
Good	25	37	24	37
Fairly good	52	49	55	50
Poor	22	15	21	13
Very poor	1	0	0	0
Grip strength (kg/cm^2^)	0.7 ± 0.3	0.7 ± 0.2	0.7 ± 0.2 ^**a**^	0.5 ± 0.2
Balance (seconds)	112 ± 34 ^**a**^	123 ± 28	120 ± 31 ^**a**^	136 ± 37
Gait velocity (m/s)	1.5 ± 0.4 ^**a**^	1.6 ± 0.5	1.4 ± 0.3 ^**a**^	1.7 ± 0.4

Analysis of the women in the same area in 1988/89 and 1998/99.

^**a**^ p<0.05 after Bonferroni correction.

Data are presented as means ± SD or as proportions (%) of valid measurements except for alcohol (g/week), which, due to non-normality, is presented as median with first and third interquartile values.

The individuals who attended both measurements had a lower mean age (61 (60, 62) years vs. 69 (68, 70) years) and higher BMD (474 (463, 486) mg/cm^2^ vs. 432 (420, 437) mg/cm^2^) at the baseline evaluation compared to those only attending the first measurement. However, when we adjusted for age differences, no difference in BMD remained between those who attended both measurements and those who only attended the first measurement (451 (441–460) vs. 453 (443–463)). We found the same when we examined the rural and urban women separately (data not shown).

## Discussion

The recently reported decrease in hip fracture incidence in many settings (Melton et al. 1996, Rogmark et al. 1999, Lofman et al. 2002, Jaglal et al. 2005, Kannus et al. 2006, Nymark et al. 2006, Chevalley et al. 2007, Icks et al. 2008) could not be verified in our cohort of Swedish women from urban and rural areas. We did not find any differences over time regarding BMD or the prevalence of osteoporosis; thus, the net effect of secular changes in other risk factors ought to be low.

Use of T-score to visualize the absolute differences in BMD makes them more applicable to the clinical situation, as a decrease of 1 SD at the distal radius by SPA has been predicted to give a 50–60% increase in the risk of hip fracture (Cummings et al. 1990, 1993, Stone et al. 2003). The absolute differences in T-score between the cohorts measured in 1988/89 and 1998/99 were small and were not statistically significant in both the rural cohort and the urban cohort. Thus, if one only takes changes in BMD into account, there ought to have been a relatively unchanged incidence of hip fracture during the study period.

The proportion of hip fractures attributable to osteoporosis has been calculated to be only 20–30% (Stone et al. 2003), and a recent report has even proposed that the burden of low trauma fractures is not related to the prevalence of osteoporosis but to a more comprehensive assessment of fracture risk (Langsetmo et al. 2008). These observations highlight the importance of evaluating other risk factors apart from BMD. Low weight and low BMI are well-known risk factors (Cummings et al. 1995, De Laet et al. 2005), but we did not find any changes in these factors over time. Another important risk factor is the risk of falling. Recent publications have actually suggested that we should shift our attention in fracture prevention from osteoporosis to reduction of the number of falls (Jarvinen et al. 2008)—or at least to consider both (Langsetmo et al. 2008). In our secondary analysis, we did not find any differences in reported fall incidence even though both balance and gait velocity were better at the second measurement in 1998/99, indicating a possible influence on fall risk.

As we anticipated, in 1998/99 there was an increased proportion of women who had ever used estrogen therapy and/or oral contraceptives compared to 1988/89. Interestingly, balance was better and gait velocity faster in both urban and rural women at the second measurement—as was the proportion of women consuming alcohol, all 3 of which could possibly have affected the number of fractures, but in different directions. In addition, rural women generally had lower grip strength in 1998/99 than in 1988/89. We did not evaluate changes in other risk factors for hip fracture such as bone structure, vision, prevalence of hip arthroplasties, and prophylactic fracture treatment (Cummings et al. 1995, Kannus et al. 1996), and we only evaluated some medications and co-morbidities.

The strengths of this study are the use of a population-based cohort for both BMD and fracture evaluation and the fact that changes in BMD and hip fracture incidence over time were estimated in the same populations. The BMD measurements were all done using the same bone scanner operated by the same technician throughout the study. The prospective evaluation of scanner drift is also another strength of the study. Furthermore, incidence of hip fracture was not presented in 5- or 10-year age classes, a study design that could be biased by changes in demographics within the age classes. Instead, we used an age-adjustment by direct standardization, thus compensating for changes within the age classes. Fracture data were collected from a complete national database in which every inpatient record is linked back to the home municipality, thus enabling us to find virtually all patients with a hip fracture, even if the fracture was sustained and treated away from the home municipality. Also, by using both a diagnosis code and an operation code for the inclusion, we improved our ability to catch only acute hip fractures within the cohorts by excluding patients with complications that were secondary to an earlier hip fracture.

One weaknesses of the study was the BMD evaluation in the forearm by the SPA technique, a clinical scanning technique that is no longer used today. Fracture prediction from BMD has been investigated more extensively by dual-energy X-ray absorptiometry (DXA), where a decrease of 1 SD in the hip has been calculated to give a 3-fold increase in the risk of hip fracture (Marshall et al. 1996) compared to SPA. With SPA, a 1-SD reduction in forearm BMD has been calculated to give a 50–60% increase in hip fracture risk (Cummings et al. 1990, 1993, Stone et al. 2003). A high correlation has been shown between BMD values measured with SPA in the distal radius and those measured with DXA in the hip (Karlsson et al. 1993), and both have been shown to be predictors of hip fracture (Cummings et al. 1993, Marshall et al. 1996, Stone et al. 2003). Ideally, the second measurement should have been done after a separate randomization, but no differences in age-adjusted BMD were evident between those attending both measurements and those attending only the initial measurement. Mean age in the 4 cohorts was similar, and therefore no age adjustment for BMD was done. Post-hoc testing with age adjustment did not alter the results (data not shown).

It would also have been preferable to collect data for hip fracture incidence and for BMD/prevalence of osteoporosis/BMI and the secondary investigation over exactly the same years and in the same cohorts. However, the longer fracture ascertainment period and age span were chosen in order to increase our ability to detect even small changes in the incidence of hip fracture. Ideally, the rural area for fracture evaluation should have included only the municipality of Sjöbo were the BMD data were collected, but no differences in hip fracture incidence or hip fracture progression were evident between Sjöbo and the 8 surrounding municipalities that were included. We acknowledge that in spite of the fact that we increased the sample size by including 8 regions around Sjöbo when evaluating the rural hip fracture incidence, the discrepancy in changes in hip fracture incidence over time compared to a variety of other reports in western societies (Melton et al. 1996, Rogmark et al. 1999, Lofman et al. 2002, Jaglal et al. 2005, Kannus et al. 2006, Nymark et al. 2006, Chevalley et al. 2007, Icks et al. 2008) may have been due to a type II error.

To summarize, we did not find any changes in age-adjusted hip fracture incidence from 1987 to 2002 and we found no secular differences in BMD or in the prevalence of osteoporosis between 1988/89 and 1998/99. This indicates that changes in other risk factors for hip fracture such as balance and gait velocity, as found in our secondary investigation, are either of minor importance or are counteracted by changes in other risk factors.
